# ASAS-NANP Symposium: mathematical modeling in animal nutrition: harnessing real-time data and digital twins for precision livestock farming

**DOI:** 10.1093/jas/skaf138

**Published:** 2025-05-04

**Authors:** Tami M Brown-Brandl, Jian Tao

**Affiliations:** Biological Systems Engineering, University of Nebraska–Lincoln, Lincoln, NE 68583; Visual Computing and Computational Media, College of Performance, Visualization & Fine Arts, Texas A&M University, College Station, TX 77845

**Keywords:** modeling, precision animal management, technology, virtual reality

## Abstract

The increasing population and urbanization have intensified livestock production, raising concerns about sustainability, animal welfare, and disease transmission. There is a need to improve the sustainability of animal production. Precision Livestock Farming (PLF) emerges as a promising solution, utilizing sensors, Internet of Things, and data analytics to enhance animal management. Digital twins create virtual replicas of physical entities such as animals, buildings, and overall farm operations. This paper highlights the transformative potential of PLF and digital twin technology. The integration of real-time data with digital twins represents a frontier of technological innovation with the potential to transform PLF. The future of PLF looks promising with advancements in artificial intelligence, machine learning, blockchain, and augmented reality, which are expected to drive further innovations and enhance the capabilities of digital twins. In this review, we provide a brief overview of the current state-of-the-art developments in the field of precision livestock agriculture and highlight the potential for digital twin technologies to transform the agricultural sector.

## Teaser

The review paper demonstrates the transformative potential of integrating digital twin technology with real-time data from precision livestock farming sensors. The approach enhances productivity, animal welfare, and environmental sustainability by enabling precise monitoring and management of farm operations, offering a path toward more efficient and sustainable agricultural practices.

## Introduction

The increasing global population, coupled with rising wealth and urbanization, is leading to a heightened demand for animal products, especially in emerging economies. The Food and Agriculture Organization projects the global demand for animal-source foods to increase by 60% to 70% to feed a population estimated by the United Nations to reach 9.7 billion by 2050. The growing demand has already resulted in the intensification and consolidation of animal production. Over the past hundred years, such consolidation has led to a marked increase in the availability of animal-derived foodstuffs while simultaneously reducing costs (MacDonald et al., 2020; “USDA ERS - Food Prices and Spending,” 2022).

However, the consolidation of animal production has raised some serious challenges. There is growing concern among the general public about the sustainability of animal production, especially in terms of social and environmental sustainability. The public is increasingly concerned about animal welfare and wants to ensure that production animals have adequate space, enrichment, and animal care (Van De Weerd and Ison, 2019). Additionally, the consolidation of animal facilities puts increased pressure on the local landscape or substantial increases in cost to ensure that facilities remain environmentally sustainable. Another concern is the risk of disease transmission between animals and humans (Rossi and Garner, 2014). These concerns underscore the urgent need for more rigorous oversight to improve environmental monitoring, animal welfare, and disease surveillance. Additionally, the decline in the availability of labor, particularly skilled workers, increases the need for new innovative solutions. One promising solution is Precision Livestock Farming (PLF), a multidisciplinary field of research focused on the development of systems that use technological advancements to improve animal management. In addition to animal management, PLF can improve animal phenotyping.

PLF has been described as the application of sensors and data processing methods to develop systems for managing animals (Banhazi et al., 2012). Several key points of PLF include minimal interference of the animals in their environment and the collection and processing of data in real-time, which will aid animal caretakers in managing animals. PLF employs cutting-edge technology and advanced computational methods to enable the constant, automated, and instantaneous monitoring of individual animals, thus optimizing animal care and facility management (Berckmans, 2017). Ongoing research into PLF technologies is exploring the use of both wearable and fixed sensors, with the goal of facilitating real-time management decisions. The research is paving the way for the integration of digital twin technology, which creates virtual replicas of animals, farm operations, and ecosystems. By simulating and analyzing these digital twins, farmers can optimize farm management, leading to improved productivity, animal welfare, and environmental sustainability. Digital twin technology is revolutionizing agriculture by providing a virtual representation of physical entities, which enables real-time monitoring, simulation, and optimization of farming practices (Grieves, 2005; Jo et al., 2018; Liu et al., 2021; Neethirajan and Kemp, 2021; Purcell and Neubauer, 2023; Engineering et al., 2024). For PLF, digital twins offer a comprehensive view of health and behavior, allowing farmers to detect early signs of illness, optimize nutrition, and improve welfare through predictive analytics. This proactive approach minimizes disease outbreaks and enhances overall productivity. By integrating with Internet of Things (IoT), artificial intelligence (AI), and big data, digital twins support precision agriculture, enabling data-driven decision-making that improves operational efficiency, profitability, and sustainability, ultimately leading to a more resilient future (Jo et al., 2018; Neethirajan and Kemp, 2021; Verdouw et al., 2021).

## PLF : An Overview

The overall goal of PLF is the application of technology to aid in the identification of individual animals in need of management intervention, for example, identifying animals ready to be marketed or finding animals that are sick. To achieve this goal, researchers need to understand the production system, animal physiology, and behavior; the technology to be applied and the analysis tools to process the data are all critical in creating a PLF system. In order to truly create new PLF applications, collaboration between animal scientists, physiologists, veterinarians, ethologists, engineers, computer scientists, and more is required (Berckmans, 2017).

While we commonly think of PLF as a modern-day solution for livestock management, the concept has a much richer history. The management of animals using computer-based methods was first introduced in 1975, with a doctoral dissertation entitled “Milk Yield as a Factor in Dairy Herd Management by Exception” (Hyde, 1975). The goal of this dissertation was to develop a computer-based system for the management of a dairy herd. This dissertation work was completed in the midst of the first development of the microprocessor in the early 1970s and the first microcomputer in 1975 (Abbate, 1999), while the average dairy herd size was under 30 cows per farm (MacDonald et al., 2020). In 2017, the average dairy herd was 175, while 16.4% of the industry’s total milk was generated from herds greater than 5000 cows. The largest farm today is over 134,000 cows located in China. Several large farms in the United States have over 15,000 cows. While the aim of the system has not changed from 1975 to today, the increase in animal numbers and the vastly superior computing power of today’s computers allow for PLF systems to incorporate much more complex systems of sensor inputs and algorithms.

**Figure 1.**
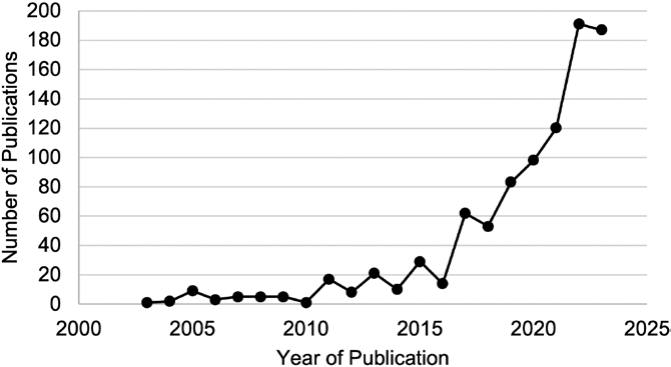
The publication record of articles with the keyword “Precision Livestock Farming.” The first PLF conference was held in 2003; however, it took several more years before the name started being used regularly.

PLF as a term was first used in a publication in 2005 by Christopher Wathes. The first PLF conference was held in 2003 in Berlin, Germany (Wathes, 2003; Werner and Jarfe, 2003). After this term was coined and started to become mainstream, it has become much easier to track the scientific effort in this field. The term has continued to gain popularity; a marked increase occurred after 2011. Figure 1 illustrates the number of publications per year using the term PLF. The Scopus database was search using the term of “Precision Livestock Farming” within the title, abstract, or keywords. Two subsequent searches were conducted using an additional term of dairy or swine.

Since the first PLF conference in 2003, researchers have used technologies on all different livestock, poultry, fish, and even insect species. The most developed PLF applications have been the dairy industry, as the animals have the highest value and are in the production cycle for a relatively long period. The swine industry has been the second most popular species for PLF applications. A total of 313 PLF focused articles have been published on dairy since 2003, compared with 202 articles published on PLF application on swine.

## Real-Time Data in Livestock Farming

Technologies used in PLF applications can be divided into four types: images, sound, tracking, and wearable/implantable (Andonovic et al., 2018).

### Images

Image-based technologies in PLF involve the use of cameras and computer vision systems to monitor livestock (Condotta et al., 2018, 2019; Pi et al., 2023; Bello and Olubummo, 2024; Guarnido-Lopez et al., 2024). Several image data types are commonly used in PLF applications: digital, depth, and thermal. Digital cameras are the least expensive of the three types of cameras. These cameras can be stand-alone cameras or part of a prepackaged surveillance camera system. The cameras can store either video or single images in either grayscale (one channel) or in color (red, green, and blue channels). Digital cameras have been used for many different applications, including weight estimation, condition score, lameness, and various well-being assessments. The disadvantage of digital images is that when automated processing is applied, lighting, different backgrounds, color differences, and patterned or dirty animals create challenges.

Depth cameras are specialized cameras that capture the distances from the camera to each pixel in the image. The output image is the surface shape instead of a visual image, as with a digital camera. There are three different technologies that capture depth images: structured light, time of flight, and stereoscopic (Condotta et al., 2020). There are cameras that also combine two of these technologies to capture depth images. The advantages of these cameras are the color patterns, backgrounds, and lighting issues are eliminated. The costs of depth cameras are relatively low. However, most of the time, each depth camera requires a single computer to capture images, which substantially adds to the cost and complexity of the system. Images captured using depth cameras have been used to estimate weight, condition score, lameness, postures, and various well-being measurements.

The third type of camera that can be used in PLF applications is a thermal camera. Thermal cameras capture temperature at each pixel instead of color or depth. The critical component for any camera is the resolution. Resolution, or the number of pixels in the horizontal and vertical directions (i.e., 60 × 80 or 320 × 480), needs to be carefully considered in thermal cameras as some of these cameras have very low resolution. Thermal images have been used to capture changes in blood flow to the skin surface, which has been used to quantify thermal comfort (Brown-Brandl et al., 2023), injuries (Stokes et al., 2012; Renn et al., 2014), infections (Racewicz et al., 2018), and pain (Whittaker et al., 2023). Body temperature is not well correlated to surface temperature captured by thermal cameras (Giro et al., 2019).

### Sound

Capturing and analyzing sound has proven to be an efficient and versatile method for gathering information from groups of animals in PLF. Sound monitoring offers numerous advantages: it is contactless, independent of lighting conditions, relatively inexpensive, not particularly sensitive to temperature variations, and applicable in both indoor and outdoor environments. These attributes make sound an appealing tool for livestock monitoring, particularly in situations where other technologies might face challenges, such as low light or extreme temperatures.

When implementing sound-based monitoring systems, several equipment considerations must be addressed to ensure reliable data collection. These include selecting an appropriate power source to support continuous operation, optimal placement of microphones to minimize interference from background noise, and determining the sampling rate, resolution, and frequency range of interest (Berckmans, 2017). The frequency range is particularly critical, as it must align with the species-specific vocalizations and sounds relevant to the monitoring objective.

Sound has been successfully applied to assess a variety of physiological and behavioral parameters in livestock. In poultry, for instance, sound has been used to evaluate thermal comfort (Moura et al., 2008) and estimate feed intake (Aydin et al., 2014). In swine, it has been employed for detecting illnesses such as respiratory conditions, which produce characteristic coughing sounds (Berckmans, 2017), as well as monitoring oestrus through vocalizations (Rhim et al., 2008) and assessing thermal comfort through stress-related vocal patterns (Hillmann et al., 2004). Sound analysis has also been extended to broader well-being assessments, capturing indicators of stress, pain, or social interactions (Nan et al., 2022).

The potential applications of sound monitoring in PLF continue to grow, driven by advances in AI/ML and signal processing. These technologies enable the development of algorithms that can automatically classify and interpret animal vocalizations and other sounds, making sound-based monitoring systems increasingly robust and user-friendly. By integrating sound data with other PLF technologies, producers can gain a more comprehensive understanding of animal health, behavior, and environmental conditions, ultimately improving management practices and animal welfare.

### Tracking

Radio-frequency identification (RFID) is a technology used to identify and track individual objects, such as persons, animals, or other items, using radio waves and electromagnetic fields. RFID systems have three components to the systems: 1) tags or transponders on the objects to be identified, 2) an antenna, and 3) a reader that activates the antenna and decodes the signal received to a tag number (Brown-Brandl et al., 2019). Each tag has a microchip with a unique serial number and an antenna for receiving power and transmitting information. RFID technology uses electromagnetic fields to interrogate the transponder. There are two different types of RFID: passive and active systems.

The passive systems, most common in livestock, rely on the antenna to activate the tag, so there is no battery within the tag itself, and therefore, the tag can only be read when in the vicinity of an antenna. Passive RFID systems operate within three primary frequency bands. Low-frequency (LF) RFID spans from 125 to 134.2 kHz and is typically employed for animal identification as per ISO 11784 and 11785 standards (Eradus and Jansen, 1999). High-frequency (HF) RFID operates at 13.56 MHz and is commonly found in smart cards, tickets, libraries, and similar applications (Chawla and Ha, 2007). Ultra-high-frequency (UHF) RFID covers the widest range, operating between 860 and 960 MHz. Different regions have adopted specific frequency bands for UHF systems, with notable ranges being 865 to 868 MHz and 902 to 928 MHz. The range 865 to 868 MHz is prevalent across Europe, Russia, Greenland, the Middle East, India, New Zealand, most of Africa, and parts of Asia, Central America, and South America. The latter range is utilized in North America, China, Australia, South Africa, and most South America (Repec, 2019). All three frequencies ranges (LF, HF, and UHF) have been used in animal agriculture (Brown-Brandl et al., 2019). There are advantages and disadvantages to each of these frequencies; most notably, UHF tags can be read at a greater distance, UHF and HF tags have anti-collision protocol, so multiple tags can be read at the same time, and LF signals are not influenced by water such as UHF and HF signals.

Global positioning devices (GPS) and ultrawide-band or active RFID (UWB) systems can track animals moving through an environment. GPS works by triangulating signals from multiple satellites to determine the precise location of a receiver on Earth. These sensors work well when applied to extensively raised livestock such as cattle or sheep (Hofstra et al., 2022). UWB tags transmit short-duration pulses of radio waves to determine their precise location relative to UWB anchor points (Porto et al., 2014). Generally, GPS works best when applied outdoors, although it has been shown to work inside with repeaters. UWB is not ideal for super large areas due to its reliance on short-range communication. The maximum range has been documented at about 200 m in ideal conditions; however, a good working range is less than 30 m (Hindermann et al., 2020). Both GPS and UWB systems collect positional data throughout the day; resulting location data can be summarized into time budget on location, distance traveled, number of traveling events, velocity, and more. Both sensors require battery power; therefore, the cost and weight are considerably more than passive RFID tags.

### Wearable or implantable

Wearable or implantable sensors have been used to monitor feeding, rumination, oestrus detection, health monitoring, body temperature, calving predication, grazing behavior, heart rate, and lameness (Barwick et al., 2018; Alipio and Villena, 2023). Implantable sensors in livestock are difficult in practice due to the potential risk of entering the food chain. Several types of wearables and rumen bolus have been developed for livestock species and have been primarily applied to cattle. A variety of sensors have been incorporated into rumen boluses to provide long-term identification, and monitor pH, rumen temperature, and activity of the animal (Hajnal et al., 2022).

Wearable sensors are designed to be attached externally to animals and are commonly placed on ear tags, collars, or legs. These sensors leverage microelectronics and wireless communication advancements to collect and transmit data efficiently. Wearable sensors are commonly applied to an ear tag, a collar, or the lower leg. These sensors have been used for activity monitoring, location, identification, drinking, eating, rumination, and others (Lee and Seo, 2021).

To monitor activity or behavior, some wearable sensors include accelerometers and gyroscopes. These sensors are integrated into wearables to provide insights into animal activity levels, movement patterns, and resting times, and changes in these factors are critical for identifying abnormal behaviors related to illness, injury, or stress (Munoz Poblete et al., 2018; Riaboff et al., 2022; Mladenova et al., 2024).

### Feed and water

Feed and water are critical elements to consider for animal health and well-being. There are several commercial systems that can measure the feed and water intake of individual animals (Hyun and Ellis, 2001; DeVries et al., 2003; Chapinal et al., 2007). In order for these systems to accurately monitor intake, the feeders are single-animal feeders and limit access to prevent multiple animals from accessing the feed at the same time. In addition, these systems tend to be expensive and require maintenance to ensure their accuracy. Several research teams have designed and built systems to monitor only the feeding/drinking behavior (Brown-Brandl, 2011; Maselyne et al., 2014; Adrion et al., 2018a). This information has been used to detect illness (Brown-Brandl et al., 2016; Kapun et al., 2016; Adrion et al., 2018b), determine impacts of management schemes (González et al., 2008; DeVries, 2019), and detect estrus (Dolecheck et al., 2015). The most common systems are based on RFID systems. Locating the antenna within the feeding space, so individual animals can be identified, and the time can be tracked.

### Understanding data needs

**Figure 2.**
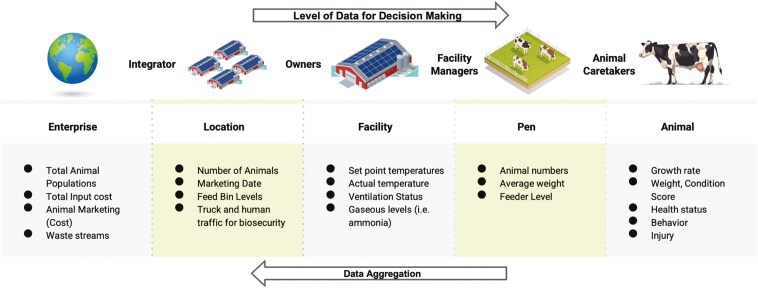
Examples of monitoring systems needed for each level and the career pathways in need of the information.

When a PLF system is being considered, careful consideration of the parameter to be measured must be made to provide information to the intended audience. Different information is needed for different levels and types of engagement with the animals (Figure 2). For example, the integrator needs to know information on the inputs and outputs to each operation (approximate animal numbers, approximate marketing dates, feed cost, etc.). In contrast, the animal caretaker would benefit from knowing individual animal information such as growth rate, individual weights/condition scores, behavior issues such as tail biters, or aggressive animal in the herd. For the most part, current systems monitor items for the enterprise, location, and facility level—and systems for the pen and animal level are under development and are the focus of PLF research. When PLF is applied to individual animals and care is managed on the individual level, animal welfare should be improved. This information should also add knowledge to the enterprise, lowering the treatment cost and allowing for more targeted selling of the animals to increase the bottom line for the enterprise.

### Transforming data to actionable information

In the realm of livestock farming, the advent of advanced sensing technologies has led to the accumulation of vast amounts of data. However, the true value of these data lies in its transformation into actionable information that can drive decision-making and optimize farm operations. This section explores the methodologies and processes involved in converting raw data into meaningful insights that can enhance livestock management, productivity, and sustainability.

**Figure 3.**
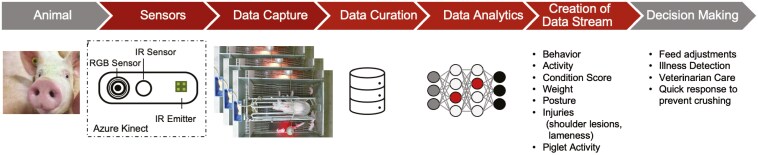
Process of creating a precision livestock farming tool from animal to decision-making. The process includes sensor selection and application, data capture and curation, data analytics, or translating the data into usable data stream, then the data stream needs to be translated into actionable information.

In order to complete this transformation, there are several steps that need to be taken (Figure 3). First, careful evaluation needs to be completed to determine what type of information would be useful to make decisions. For example, to determine illness, there are several different data types that could be used, including images, sound, RFID data (to determine presence at the feeder/drinker), body temperature, and maybe more. This decision needs to be carefully considered to 1) determine what information can be obtained, 2) how it interferes with the animals, 3) whether the information is accurate, 4) whether it captures individual or group information, etc. There are other considerations that need to be considered including cost, installation requirements, maintenance, and durability.

Once the sensor type is selected, then the frequency of capture needs to be determined. According to the Nyquist–Shannon sampling theorem, samples should be collected at a minimum of 2 times the frequency of occurrence (Por et al., 2019). However, it is common to use a sampling frequency of 10 times the frequency of occurrence to ensure optimum event capture (Raju et al., 2023). For example, if measuring diurnal temperature, there is a complete cycle within 24 h, so data should be collected at a minimum of two times per day. However, if it is important to understand the peak amplitude of the signal, it is important to sample the signal at least 10 times the frequency of the highest frequency of interest. For example, if you would like to know the maximum temperature over the day, then you need to sample at 10 times per day. Data can be oversampled to improve single resolution, reduce noise, ease data filtering, and reduce the risk of misinterpreting the data frequencies. In addition, biological data and weather parameters are not simple sinusoidal waveforms, so faster sampling frequencies will enable the capture of small details. The same principles can be applied to behavior sampling, as well. Therefore, if an eating event takes an average of 10 min with durations between 3 and 30 min, the behavior data should be captured at a minimum of every 90 s (3 min divided by 2 samples per occurrence) and ideally, it should be captured every 18 s (3 min divided by 10 samples per occurrence).

While it seems like a simple solution is to capture data at the fastest possible frequency, the time and cost involved in data curation make the selection of data sampling frequency important. Data curation is a crucial process that includes organizing, maintaining, and preserving data to ensure its quality, accessibility, and usability over time. With proper data curation, the value of data is enhanced by making it more reliable, discoverable, and reusable for future research and decision-making. Data curation involves careful consideration of data integrity, including validation, cleaning, and documentation, to ensure accuracy and consistency. In addition to the data files, metadata, which provides context and details about the data, needs to be carefully documented and stored with the data, making the data easier for others to understand and utilize. Ethical considerations, such as privacy and data ownership, must also be addressed to protect sensitive information and comply with legal and ethical standards. Additionally, data curation supports long-term preservation, enabling data to be stored securely and retrieved when needed, thus ensuring that valuable datasets remain accessible for future use. In an era of big data and open science, effective data curation is essential for maximizing the impact and sustainability of research.

Data analytics must be used to transfer the data from its raw form to a usable data stream. Several different types of algorithms have been used to process raw data. For example, RFID data collected at the feeder needs to be filtered and processed so meal parameters and total time can be determined. Another example is to determine the postures of behaviors from images or videos. Different algorithms can be applied, from simple mathematical solutions to deep learning algorithms. Once this transfer is made, the information can be combined to create a data stream or a continuous flow of data ordered in a time sequence.

Once the raw data has been preprocessed into a data stream, different modeling techniques need to be used to transform the data into actionable information. These approaches are different from statistics that are applied to determine the significance between treatments, as these approaches determine the probability of differences based on means and standard deviations. The focus for PLF applications is on individual animal information. For example, using anomaly detection changes in feeding behavior can be used to predict animal illness, using the feeding behavior of single animal changes over time. In other cases, images that have been converted to area or volume need to be converted to weight.

## Digital Twins in Livestock Farming

**Figure 4.**
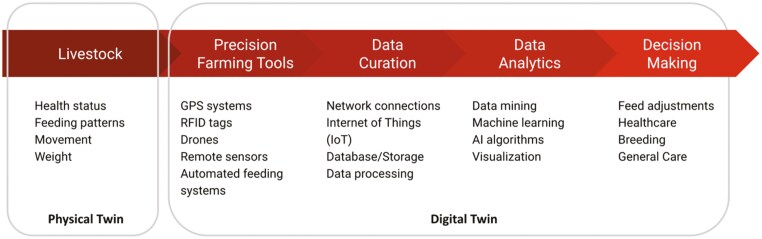
The overview diagram of a digital twin for livestock farming. It shows how data about livestock are collected using precision farming tools and processed through data curation and analytics to enhance decision-making in areas such as feed adjustments, healthcare, breeding, and general care.

Digital twins are virtual replicas of physical entities, processes, or systems, and in PLF, they represent individual animals, herds, or entire farming operations points (Neethirajan and Kemp, 2021). These digital models are created using data from sensors, cameras, and other monitoring devices, enabling real-time monitoring of livestock health, behavior, and environmental conditions. By analyzing historical and real-time data, digital twins offer predictive analytics to foresee potential health issues, optimize feeding schedules, and improve overall farm management (see Figure 4). They also allow farmers to simulate different scenarios and management strategies, leading to optimized resource use, enhanced animal welfare, and increased productivity. Integrating data from various sources provides a comprehensive view of farm operations, helping identify patterns and correlations that might not be evident from isolated data.

### Concept of digital twins

The idea of creating virtual models of physical objects can be traced back to the early days of computer-aided design and computer-aided engineering in the 1960s and 1970s. These early systems allowed engineers to create digital representations of physical objects for design and analysis purposes. The term “digital twin” was first coined by Dr Michael Grieves at the University of Michigan in 2002 (Grieves, 2005). However, the practical application of the concept dates back to NASA’s Apollo program in the 1960s. NASA used paired physical and digital systems to simulate and predict the behavior of spacecraft, which was crucial for mission success and safety (Grieves, 2005). The concept of digital twins represents a groundbreaking technological paradigm that has been gaining significant traction across various industries, fundamentally transforming how businesses operate, innovate, and deliver value to their customers (Fuller et al., 2020; Liu et al., 2021; Sharma et al., 2022; Yao et al., 2023). At its core, a digital twin is a virtual model designed to accurately reflect a physical object, process, system, or service. This digital replica is not a static representation but a dynamic and evolving entity that is updated with real-time data collected from sensors embedded in the physical counterpart. This continuous flow of data enables the digital twin to simulate the current state of its physical twin accurately, predict future states, and look back at past states, thus providing a comprehensive temporal view of the object.

While the inception of digital twins was in early 2000, it was not until the advent and proliferation of the IoT technologies that the concept truly began to flourish and expand its applicability across a myriad of sectors, including manufacturing, healthcare, urban planning, agriculture, and more (Schluse et al., 2018; Liu et al., 2021). The IoT serves as the nervous system of the digital twin, providing the sensory data needed to feed the virtual model with information about the physical world. This symbiotic relationship between the digital and physical realms opens up unprecedented opportunities for analysis, diagnostics, and prognostics in real-time (Sharma et al., 2022).

One of the most compelling aspects of digital twins is their ability to serve as a sandbox for experimentation and simulation (Schluse et al., 2018). By leveraging the power of advanced analytics and AI/ML, digital twins allow for the exploration of “what-if” scenarios without the risk of disrupting the actual physical system. This capability is invaluable for optimizing performance, anticipating potential failures, and testing new strategies or designs before they are implemented in the real world. For instance, in the manufacturing sector, digital twins of production lines can be used to identify bottlenecks, predict maintenance needs, and improve overall efficiency, thereby reducing downtime and increasing productivity (Liu et al., 2021).

Digital twins also play a crucial role in the realm of predictive maintenance, a strategy that aims to predict when equipment or systems will fail or require maintenance, thus preventing unexpected breakdowns and extending the lifespan of assets (Aivaliotis et al., 2019). By continuously monitoring the condition and performance of physical assets, digital twins can alert operators to anomalies that may indicate impending failures, allowing for timely interventions that can save both time and money (Liu et al., 2021; Yao et al., 2023).

Despite the vast potential of digital twins, their implementation is not without challenges. The creation and maintenance of a digital twin requires significant investment in technology and expertise, including the deployment of IoT sensors, data storage and processing infrastructure, and advanced analytical tools. Additionally, concerns regarding data privacy and security must be addressed, particularly when dealing with sensitive information (Aivaliotis et al., 2019).

### Digital twin’s relevance to livestock farming

Digital twins are increasingly being adopted in livestock farming to enhance animal welfare, optimize operations, and improve decision-making (Jo et al., 2018). Within the framework of PLF, digital twins integrate biometric sensors and big data analytics to monitor health and behavior of a wide variety of farm animals in real-time, enabling informed decision-making and proactive disease prevention (Neethirajan and Kemp, 2021). As part of the SmartAgriFood and Fractals accelerator projects, digital twins were employed to monitor the activity of dairy cows using advanced pedometer systems (Verdouw and Kruize, 2017). These pedometers tracked movement patterns, which are critical for identifying estrus, a period when cows are most fertile and ready for breeding. By detecting increased activity levels associated with estrus, this technology enabled precise timing for artificial insemination, thereby improving reproductive success rates. Such real-time monitoring not only enhanced breeding efficiency but also reduced labor-intensive practices such as manual observation, allowing farmers to optimize herd management and maximize productivity.

#### Monitoring and managing individual animal health

At the heart of digital twin technology in livestock farming is the ability to monitor and model the health, behavior, and productivity of each animal individually (Jo et al., 2018; Neethirajan and Kemp, 2021; Peladarinos et al., 2023). By outfitting livestock with IoT sensors, farmers can collect real-time data on vital signs, movement, feeding patterns, and more. These data feed into the digital twin, which mirrors the physical state of the animal in real-time, allowing for immediate analysis and action.

Through image and sound analysis, they also create digital representations of animals to monitor behaviors that may indicate stress or health issues, facilitating timely interventions to enhance animal welfare in poultry and pig production (Norton et al., 2019). The advent of image analysis in the early 1990s marked a pivotal moment in livestock monitoring (Van der Stuyft et al., 1991). Over the years, this technology has been harnessed to evaluate various aspects of animal health and development. For instance, it has been used to estimate weight, assess mobility issues in poultry, track water consumption in pigs, and even recognize individual pigs within crowded enclosures (Schofield et al., 1999; Aydin et al., 2010, 2014; Kashiha et al., 2013a, 2013b).

Moreover, digital twins integrate data from wearable devices such as electronic ear tags and GPS collars to assess habitat selection, movements, health, and welfare in large pastures. Automated tracking systems using depth video cameras further enhance monitoring by continuously tracking activities such as feeding and drinking, which is crucial for early health issue detection (Matthews et al., 2017). Additionally, virtual fencing technology powered by digital twins enables precise grazing management by containing livestock within designated areas using audio cues and mild electric pulses. This approach optimizes grazing patterns while ensuring animal welfare (Herlin et al., 2021).

#### Optimizing farm operations and management

Beyond individual animal health, digital twins facilitate the optimization of overall farm operations. They can simulate different management strategies, feed regimes, and environmental modifications to predict their impacts on animal welfare, productivity, and resource utilization. This facilitates population-level analyses that improve decision-making and operational efficiency (Neethirajan and Kemp, 2021). In disease control, digital twins simulate livestock environments to identify and mitigate the spread of diseases. These virtual simulations enable testing of interventions in a controlled setting before real-world application, reducing costs associated with disease outbreaks (Jo et al., 2018). Furthermore, digital twins are integral to PLF, leveraging biometric sensors and big data analytics to monitor animal health and behavior in real time.

#### Environmental impact management and sustainability

Digital twins also offer a powerful tool for managing the environmental impact of livestock farming. By enabling real-time monitoring and control, digital twins contribute to reducing greenhouse gas emissions by optimizing animal production processes and resource use, a key feature of PLF technologies (Tullo et al., 2019). On the sustainability front, digital twins optimize resource use, such as water, feed, and energy, by providing detailed insights into farm operations, thus fostering more sustainable practices (Ametefe et al., 2024).

### Creation and maintenance of digital twins

Digital twins are not merely virtual models but dynamic, data-driven representations of physical systems, created by synthesizing input from sensors, IoT devices, and historical records. These models serve as comprehensive digital replicas of target objects and their environments, enabling real-time interaction and analysis (Ferko et al., 2022; Segovia and Garcia-Alfaro, 2022). In the context of PLF, this process leverages advanced AI/ML techniques to continuously process and analyze data streams, offering actionable insights into animal behavior, distress signals, and disease patterns. By integrating modeling and simulation technologies, digital twins replicate real-world farm conditions with high fidelity, allowing farmers to test hypothetical scenarios and predict outcomes to refine management strategies. Maintenance of digital twins hinges on real-time synchronization with physical systems via IoT devices, ensuring the virtual model remains an accurate reflection of its physical counterpart (Neethirajan and Kemp, 2021). Predictive analytics is another key component, helping foresee issues such as disease outbreaks or behavioral changes in livestock, thereby mitigating risks and optimizing operations (Neethirajan and Kemp, 2021).

#### A conceptural architecture for digital twin development in PLF

**Figure 5.**
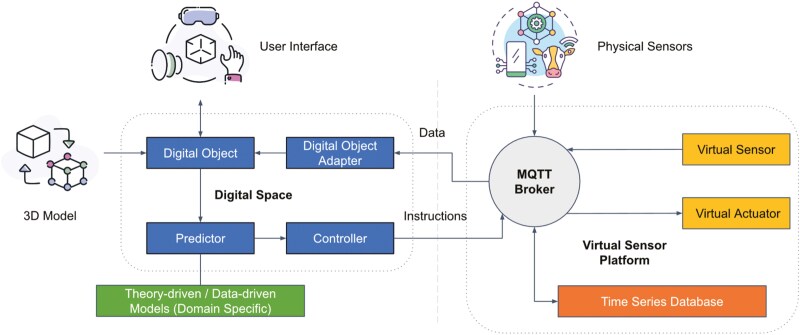
Overview architecture of a sample digital twin development platform, illustrating the integration of 3D models, digital objects, user interfaces, and physical sensors. The system features components such as a Predictor, Controller, Digital Object Adapter, MQTT Broker, Virtual Sensor Platform, Virtual Sensors, Virtual Actuators, and a Time Series Database, highlighting the data flow and interaction between virtual and physical elements.

To advance understanding in this domain, a conceptual architecture for digital twin development in PLF is proposed. A digital twin development platform is presented, as illustrated in Figure 5, providing a framework that delineates the sequential processes essential for creating and operating a digital twin. The proposed architecture outlines the integration of key components, including data acquisition, modeling, simulation, and real-time monitoring, emphasizing their interconnectivity in forming a cohesive and functional system. By incorporating this architecture, the discussion clarifies the step-by-step processes critical for the development and maintenance of digital twin platforms, offering valuable insights into their structural and operational dynamics.

#### Virtual replica

At its core, the framework integrates a 3D model that serves as the virtual counterpart of a physical asset or system. This digital object resides within a digital space that includes a *Predictor* and a *Controller*. The Predictor utilizes data-driven models (e.g., AI/ML algorithms) or theory-based models to make accurate predictions about the physical asset’s behavior. The Controller, on the other hand, sends instructions back to the physical twin based on these predictions. The Digital Object Adapter serves as an interface, ensuring seamless communication between the digital object and the data.

#### Information exchange

A key component of the framework is the MQTT (Message Queuing Telemetry Transport) Broker, which acts as a central communication hub. MQTT is an ISO (International Organization for Standardization) standard (ISO/IEC 20922:2016) publish-subscribe-based messaging protocol designed for lightweight and efficient communication, particularly within the IoT environments. An MQTT Broker facilitates message exchange between devices by receiving messages from publishers and distributing them to subscribers based on topic subscriptions. This architecture decouples message producers from consumers, enhancing scalability and reliability. As part of the sample digital twin framework, the MQTT Broker facilitates the exchange of data and instructions between the digital space, physical sensors, virtual sensors, and virtual actuators. The physical sensors collect real-time data from the physical asset, which is then processed and analyzed within the digital space. In this framework, it enables seamless data flow between physical sensors, virtual sensors, actuators, and the central processing unit, ensuring real-time monitoring and control capabilities.

#### IoT integration

After establishing the framework for the digital twin, the subsequent step is to integrate IoT sensors and devices with the physical counterpart. These sensors act as the digital twin’s sensory system, delivering a constant flow of data on parameters such as temperature, pressure, location, and movement. The selection and number of sensors deployed are determined by the complexity of the physical object and the desired level of operational detail. The digital twin evolves with every piece of new data, ensuring that it always reflects the current state of its physical counterpart. This dynamic updating process is crucial for the digital twin to serve its purpose, allowing it to simulate future states, conduct “what-if” analyses, and provide actionable insights.

#### Sample digital twin of pig farm

A digital twin of the pig farming process might involve hundreds of sensors deployed throughout the farm to monitor various aspects of pig health and environmental conditions. These sensors collect data on factors such as feed intake, weight gain, temperature, and humidity. The collected data are then transmitted to a central processing unit, where it is analyzed in real-time to update the digital twin. This virtual model allows farmers to visualize the current state of the farm, predict potential issues, and optimize operations. Smart feeders equipped with sensors can adjust the feed composition based on the pigs’ age and health status, ensuring optimal nutrition and reducing waste. Additionally, augmented reality devices can provide farm technicians with real-time data visualization and diagnostic information, enabling them to make informed decisions and perform maintenance tasks more efficiently.

#### Maintenance of digital twins

Maintaining a digital twin involves regular updates not just to its data, but also to its underlying models and algorithms. As the physical object changes, whether through aging, modifications, or repairs, the digital twin must be adjusted accordingly. This may involve recalibrating sensors, updating the digital model to reflect physical changes, or retraining ML algorithms based on new data patterns. Maintenance also includes ensuring the integrity and security of the data collected and the performance and reliability of the IoT infrastructure. This is a nontrivial task, given the potential for cyber threats and the need to comply with data protection regulations.

#### Collaboration in digital twin development

The creation and maintenance of digital twins requires a multidisciplinary approach involving expertise in fields such as computer science, engineering, data science, cybersecurity, and subject matter experts (Ferko et al., 2022; Segovia and Garcia-Alfaro, 2022). Collaboration among these disciplines is essential to address the challenges that arise at the intersection of the physical and digital worlds. Startups and the private sector play an important role in this ecosystem by driving innovation and developing commercial solutions that make digital twin technology more accessible and scalable. Startups are often at the forefront of creating specialized applications and tools that cater to niche markets. The private sector, including large corporations, provides the necessary infrastructure and investment to support these innovations, enabling the integration of digital twins into existing business processes. Commercial solutions offered by these entities can enhance operational efficiency, reduce costs, and improve decision-making, ultimately contributing to the broader adoption and success of digital twin technologies.

### Challenges and solutions

Harnessing the full potential of this synergy requires overcoming significant challenges, including the need for robust data infrastructure, advanced analytics capabilities, and stringent data security measures (Neethirajan and Kemp, 2021; Engineering et al., 2024). The vast amounts of data generated by IoT devices must be collected, transmitted, and processed efficiently and securely to ensure the accuracy and reliability of digital twins. This necessitates significant investment in technology and expertise, as well as ongoing efforts to address privacy concerns and data protection regulations.

Despite these challenges, the integration of real-time data with digital twins represents a frontier of technological innovation with the potential to transform PLF. By enabling more accurate, dynamic, and predictive analysis of the physical world, this synergy empowers organizations and individuals to make more informed decisions, optimize operations, and innovate in ways previously unimaginable.

## Impact and Future Prospects

The advent of digital twin technology is poised to revolutionize PLF, offering transformative impacts and promising prospects. As the agricultural sector increasingly embraces cutting-edge technologies, especially dairy industry, the future of PLF looks brighter than ever, with continuous advancements in AI/ML and data analytics set to drive further innovations. The potential for digital twins to foster sustainable and ethical farming practices, reduce CAE environmental impacts, and enhance overall farm management underscores their critical role in shaping the future of agriculture.

### Incorporating emerging technologies

One of the key trends in smart farming is the integration of AI and ML algorithms with real-time data analytics (Odintsov Vaintrub et al., 2021; Hurst and Spiegal, 2023). These technologies can analyze vast amounts of data to identify patterns and make predictive recommendations, further enhancing decision-making processes. For example, AI-driven predictive models can forecast disease outbreaks, optimize breeding programs, and improve genetic selection, leading to healthier and more productive livestock populations. Another emerging trend is the use of blockchain technology to enhance traceability and transparency in the supply chain. By recording every transaction and movement of livestock on a decentralized ledger, blockchain ensures the integrity and authenticity of data, fostering trust among consumers and stakeholders.

### Empowering education and workforce development

The integration of digital twin technologies in PLF is set to significantly impact education and workforce development, empowering the next generation of agricultural professionals (Hazrat et al., 2023). By combining digital twins with augmented reality and virtual reality technologies, educational institutions can offer immersive, hands-on training experiences that simulate real-world farming scenarios. This innovative approach allows students and trainees to gain practical skills and insights into livestock management without the need for physical presence on a farm. Additionally, digital twins provide decision-support tools that enhance farmers’ ability to manage livestock effectively, fostering a more knowledgeable and skilled workforce. The future of PLF will also benefit from increased collaboration and data sharing among stakeholders, facilitated by cloud-based platforms and data interoperability standards. This collaborative environment will enable the aggregation and analysis of data from multiple sources, leading to more comprehensive insights and innovations. As a result, educational programs can stay up-to-date with the latest advancements, ensuring that the workforce is well-equipped to leverage cutting-edge technologies for sustainable and efficient farming practices.

## Ethical and Regulatory Considerations

As these technologies rely on extensive data collection from sensors and IoT devices, there are legitimate concerns about how these data are handled, stored, and protected. Ensuring robust data privacy measures and security protocols is paramount to prevent unauthorized access and misuse of sensitive information (Elliott and Werkheiser, 2023; Neethirajan, 2023). Additionally, the regulatory landscape governing the use of digital twin technologies in livestock farming is evolving. Existing regulations may need to be updated to address the unique challenges posed by these advanced technologies, including data ownership, consent, and transparency. Potential regulations could mandate stringent data protection standards and establish clear guidelines for the ethical use of digital twins, ensuring that the benefits of these technologies are realized without compromising ethical standards. As the adoption of digital twins in livestock farming grows, it will be essential for policymakers, industry stakeholders, and technology developers to collaborate in creating a comprehensive regulatory framework that balances innovation with ethical and privacy considerations.

## Conclusion

Harnessing real-time data and digital twins for precision PLF has emerged as a transformative approach with far-reaching impacts on the agricultural sector. The integration of these technologies has led to significant improvements in efficiency, productivity, and animal welfare. Real-time data collection through sensors and IoT devices allows for continuous monitoring of livestock health, behavior, and productivity, enabling timely and informed decision-making. Digital twins, which are virtual replicas of physical livestock, provide detailed simulations and predictive analytics, optimizing resource utilization and enhancing overall farm management. These technologies also contribute to sustainable farming practices by minimizing waste, reducing environmental impacts, and promoting transparency and accountability through lifecycle tracking of livestock products. Furthermore, the future of PLF looks promising with advancements in AI/ML, blockchain, and augmented reality, which are expected to drive further innovations and enhance the capabilities of digital twins.

The future of real-time data and digital twins in PLF is incredibly promising, with continuous technological advancements set to revolutionize the industry. As these technologies evolve, they will empower farmers to achieve higher levels of precision, sustainability, and productivity. To fully realize the potential of digital twins and real-time data, it is essential to address ethical and regulatory considerations, particularly concerning data privacy and security. Policymakers, industry stakeholders, and technology developers must collaborate to establish comprehensive regulatory frameworks that balance innovation with ethical standards. Additionally, the integration of digital twins with augmented reality and virtual reality can provide immersive training and decision-support tools, enhancing education and workforce development in the agricultural sector. By fostering a collaborative environment and promoting data sharing among stakeholders, the agricultural industry can leverage comprehensive insights and innovations to drive sustainable and efficient farming practices. In conclusion, the adoption of real-time data and digital twins in PLF represents a significant step forward in modern agriculture, offering transformative benefits and paving the way for a more resilient and sustainable future.

## Abbreviations

AI: artificial intelligence
CAD: computer-aided design
CAE: computer-aided engineering
EDGES: Enhancing Development and Generating Excellence in Scholarship
GPS: global positioning devices
HF: high-frequency
IoT: Internet of Things
ISO: International Organization for Standardization
LF: low-frequency
ML: machine learning
MQTT: Message Queuing Telemetry Transport
PLF: precision livestock farming
RFID: radio-frequency identification
UHF: ultra-high-frequency
UWB: ultrawide-band or active RFID
